# Salvianolic acid B promotes bone formation by increasing activity of alkaline phosphatase in a rat tibia fracture model: a pilot study

**DOI:** 10.1186/1472-6882-14-493

**Published:** 2014-12-15

**Authors:** Xufeng He, Qiang Shen

**Affiliations:** Department of Traumatology; Department of dermatology, Shuguang Hospital Affiliated to Shanghai University of Traditional Chinese Medicine, Shanghai, China; Department of Orthopedic Surgery, Shanghai First People’s Hospital, Shanghai Jiao Tong University, 85 Wu Jin Road, Shanghai, 200080 China

**Keywords:** *Radix Salviae miltiorrhizae*, Salvianolic acid B, Fracture healing, Alkaline phosphatase

## Abstract

**Background:**

*Radix Salviae miltiorrhizae* is a herb frequently used within traditional Chinese medicine for the treatment of cardiovascular- and trauma-related diseases. Danshen is the dried root of Salviae miltiorrhizae, from which the polyphenolic compound Salvianolic acid B (Sal B) can be obtained. Sal B is a key component of Danshen. The aim of this study was to determine the effect of Sal B on the healing of long bones following trauma in a rat tibia fracture model.

**Methods:**

Tibia fractures were created in 20 male Sprague Dawley rats. The animals were divided into two groups: (1) experimental group (n = 10); and (2) control group (n = 10). Rats in the experimental group were intraperitoneally administered with Sal B (40 mg/kg/d) for 3 weeks, while rats in the control group received an identical volume of physiological saline solution, administered in the same way. X-ray photographs were taken of all animals at the time points. Rats were euthanized at weeks 1, 3, 8 and 12 post-fracture. Fracture calluses were measured and callus sections were obtained and stained using hematoxylin and eosin (HE) and the calcium cobalt method. HE stained sections were observed and evaluated according to different grades of bone remodeling. Sections stained using the calcium cobalt method were analyzed with an imagine analysis system.

**Results:**

Data showed that callus growth was significantly greater in the experimental group compared with the control group (P < 0.05). Furthermore, histological scores in the Sal B-treated group were statistically higher than in the saline treated group at weeks 1, 3 and 8 post-fracture (P < 0.05). Alkaline phosphatase (ALP) activity was enhanced in the experimental group at weeks 1 and 3 post-fracture (P < 0.05).

**Conclusions:**

Our results suggest that Sal B may accelerate early-stage fracture healing. Increased activity of ALP may be one factor which promotes the healing process. This pilot study provides brief insight into the effect of Sal B in fracture healing. These findings will contribute to the development of more and enhanced treatment options for trauma fracture patients.

## Background

**Figure 1:**
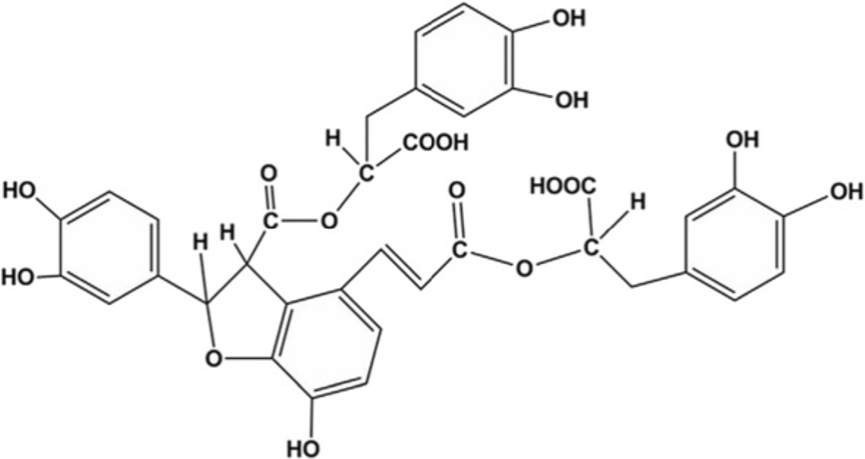
**Molecular structure of Salvianolic acid B (Sal B).**

*Radix Salviae miltiorrhizae* (Danshen) is a plant belonging to the Labiatae Lagurus grass species. It is widely used in clinical practice for the prevention and treatment of vascular diseases [1–3] and can also exert protective effects on the liver [4], kidneys [5–7] and lungs [8, 9]. Furthermore, Danshen is an effective herb within traditional Chinese medicine (TCM), commonly used for treating trauma wounds and fractures. Several studies have shown that Danshen may play an important role in accelerating bone remodeling to promote fracture healing [10, 11]. Danshen is the dried root of Salviae miltiorrhizae and can be divided more specifically into lipophilic and hydrophilic fractions [12, 13]. Salvianolic acid B (Sal B) is a water-soluble active component isolated from Danshen [12, 14, 15]. It is the main constituent of Salvia phenolic acid and the most active constituent of water-soluble salvianolic acid substances [16]. The structure of Sal B is shown in Figure 1, which consists of three molecules of Tanshinol and one molecule of caffeic acid. Its molecular formula is C_36_H_30_O_16_.

In recent years, increasing attention has been directed to Sal B, particularly within the field of cardiovascular disease [17]. Some research groups have shown that Sal B can attenuate the effect of myocardial ischemia-reperfusion injury [18] and relieve brain injury by reducing neuronal damage following cerebral ischemia [19]. As an effective component of Danshen, Sal B has been shown to prevent bone loss in prednisone-treated rats by increasing alkaline phosphatase (ALP) activity at a specific dose and time [20]. Whether Sal B can promote bone union in fractures through the same mechanism remains to be elucidated. We therefore hypothesized that Sal B would promote fracture healing by increasing ALP activity. The aim of the current study was to validate our hypothesis using a rat tibia fracture model.

## Methods

Twenty male SD rats were used in the study with approval from the Experimental Animals Ethics Committee of Shanghai University of TCM. The study was conducted at the Experimental Animals Research Laboratory of the Shanghai University of TCM, according to The Guide for the Care and Use of Laboratory Animals.

The mean age of the rats was 7 weeks and their mean body weight was 225 g. The animals were randomly divided into two groups with 10 animals in each group. None of the animals received antibiotic prophylaxis before or after the fracture and no animal was lost during the study.

The right tibias of all rats were fractured using a custom-made three-point bending device as described before [21]. Tibias were then fixed carefully using plaster casts for 4 weeks to stabilize the fractures.

The two groups were designated as the experimental group (n = 10) and control group (n = 10). On the day of operation, 10 ml of isotonic sodium chloride (0.9% NaCl) was injected intraperitoneally into the control group rats. In the experimental group rats, Sal B (purity ≥98%, Shanghai Institute of Liver Disease) was injected intraperitoneally (dissolved in 10 ml isotonic sodium chloride) at a dosage of 40 mg/kg/day after fracture formation for 3 weeks.

Rats from each group were euthanized using high-dose ketamine on weeks 1 (n = 3/group), 3 (n = 3/group), 8 (n = 2/group) and 12 (n = 2/group) post-fracture.

After euthanasia, rat right tibias were disarticulated from their ankle and knee joints. Soft tissues on the tibiae bone were gently peeled off without harm to the callus tissue. Callus diameter was measured with a vernier caliper in the sagittal and coronal planes. The diameter of each tibia, 1.5 cm proximal from the callus, was measured identically. We obtained the differences between the aforementioned diameters and carried out statistical evaluation.

All right tibias were examined radiologically to ensure the fracture had not shifted at weeks 1, 3 and 8 post-fracture. Tibias were studied radiologically at weeks 1, 3 and 8 post-fracture. Specimens were radiographed in a lateral projection using X-ray apparatus (SIEMENCE, Siremobil compact). The specimens were exposed for 2 s at 55 kV for optimal observation of the fracture status, callus formation, bony continuity and remodeling.

All calluses were also examined histologically following hematoxylin and eosin (HE) staining of sections to observe cartilage and bone formation. Specimens were then fixed in 4% buffered formaldehyde for approximately 48 h at room temperature and decalcified in 7% nitric acid (changed every 2 days) for about 7 days. Afterwards, demineralized tissues were washed, dehydrated in gradient alcohol, embedded in paraffin wax, and cut into 4 μm thick sections along the transverse section of the tibia callus. These sections were stained with hematoxylin and eosin, and the slices were examined using light microscopy. Histological grading of fracture healing was performed according to a 5-grade system as previously described [22] (Table 1).

**Table 1:** **Histological grading of fracture healing scores**

Histological evaluation	Grade
Pseudoarthrosis formation	0
Incomplete cartilaginous union	1
Complete cartilaginous union	2
Incomplete bony union	3
Complete bony union	4

Following this, sections were stained using the calcium cobalt method. In brief, specimens were deparaffinized and immersed into 10% MgCl_2_ solution for 4 hours. Specimens were then incubated at 37°C for 4–6 hours. Then, specimens were stained with solutions of 2% cobalt nitrate and 1% ammonium sulfide in turn. After being air dried, slides were finally mounted and used for microscopy. The black precipitates could be observed as the positive stained areas. Image-Pro Plus 6.0 software was used for analysis of the images. Positive stained areas were chosen, also called AOI (areas of interest), and the software was used to measure the area and obtain statistical parameters (for example, means and sum). Measurement data were then exported to an Excel spreadsheet.

The histological score were analyzed by nonparametric Manne-Whitney test with significance level set at 5% (*P* < 0.05).Other data are expressed as means ± standard deviation (SD) and analyzed using the Student’s *t* test as well as the least significant difference test by SPSS 16.0 statistical software. Assuming double-sided independent variance, *P* < 0.05 was considered statistically significant.

## Results and discussion

The weights of all rats were recorded and are summarized in Table 2. The weights and weight gains at weeks 1, 3, 8 and 12 were not significantly different between groups (*P* > 0.05).

**Table 2:** **Rat weight changes (g)**

Group	Preoperative	Week 1	Week 3	Week 8	Week 12
Saline Control	234.60 (41.41)	289.70 (42.01)	339.86 (43.59)	409.75 (116.72)	461.50 (28.99)
Sal B	217.90 (37.71)	269.60 (35.25)	325.43 (41.86)	337.75 (58.53)	339.50 (43.13)
P value	0.358	0.262	0.539	0.312	0.080

Note: Values are means (SD).

**Figure 2:**
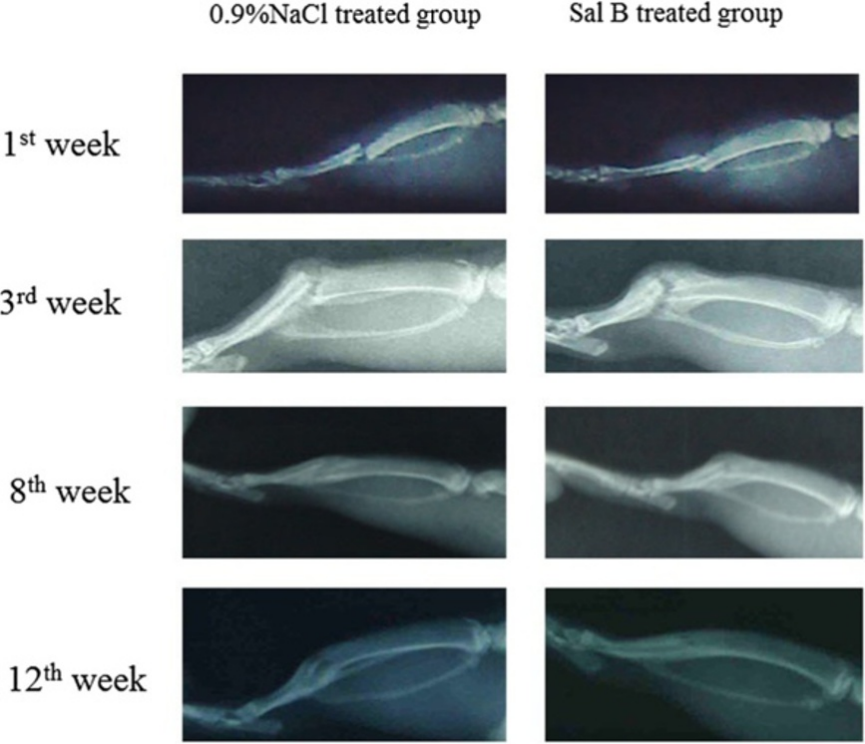
**Lateral radiographs of rat tibia were used to observe the bone fracture.** At week 3, the Sal B-treated group had a larger callus area than the saline-treated group. At week 8, the callus in the saline-treated group continued to remodel, while the Sal B-treated group showed advanced callus remodeling, indicating a faster fracture union in the Sal B-treated group.

The results of the X-ray photographs revealed that there was no fracture disunion in all tibias (Figure 2). Callus growth in the experimental group was significantly enhanced compared with the control group (*P* < 0.05) (Table 3).

**Table 3:** **Maximum callus and tibia (1.5 cm from callus) diameters (mm) in sagittal/coronal planes**

	Saline control (n = 10)	Sal B (n = 10)
Maximum callus diameters in the sagittal plane	10.11 (1.87)	11.25 (2.23)
Maximum callus diameters in the coronal plane	7.58 (1.47)	8.40 (1.06)
Diameter of tibia that 1.5 cm away from the callus in the sagittal plane	7.83 (1.18)	7.85 (1.08)
Diameter of tibia that 1.5 cm away from the callus in the coronal plane	6.08 (1.04)	6.12 (0.72)
Differences between diameters in the sagittal plane	2.28 (0.88)	3.40 (1.27)^a^
Differences between diameters in the coronal plane	1.50 (0.64)	2.28 (0.85)^b^

Note: Value are means (SD), a:P = 0.034, b:P = 0.033.

**Figure 3:**
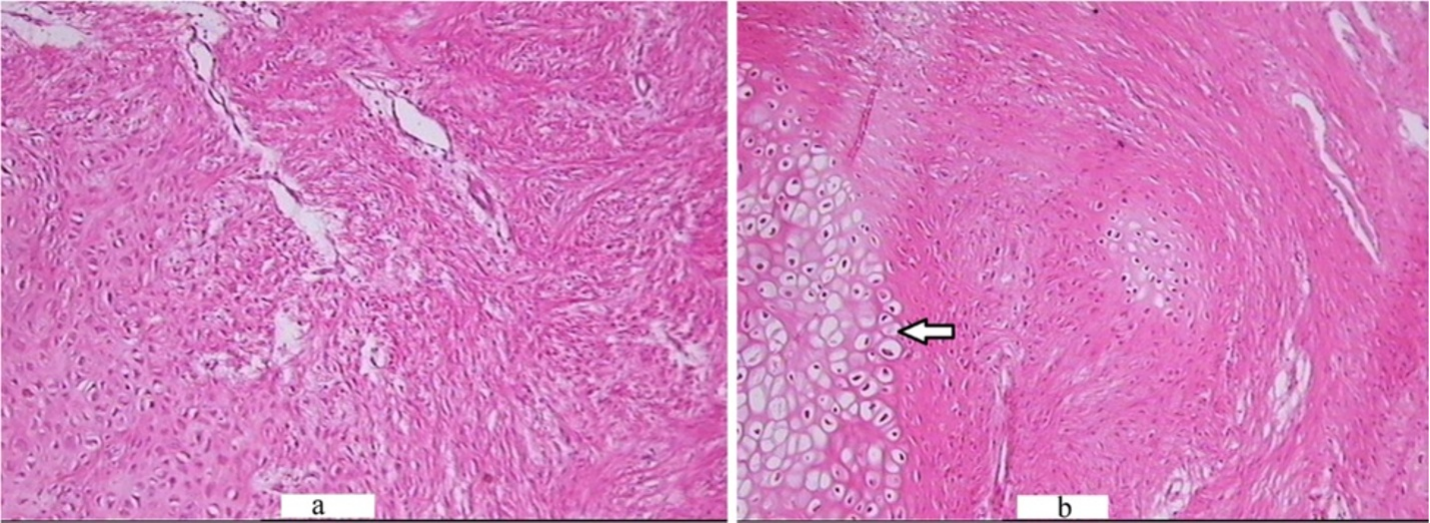
**HE staining of the callus in the saline-treated (a) and Sal B-treated (b) groups at week 1 post-fracture. (a)**: Abundance of fibroblasts with few cartilage cells (which could secrete ALP) were observed in the saline-treated group at week 1 post-fracture (HE × 100) **(b)**: A greater number of cartilage cells (arrow) were observed in the Sal B-treated group at identical time points (HE × 100).

**Figure 4:**
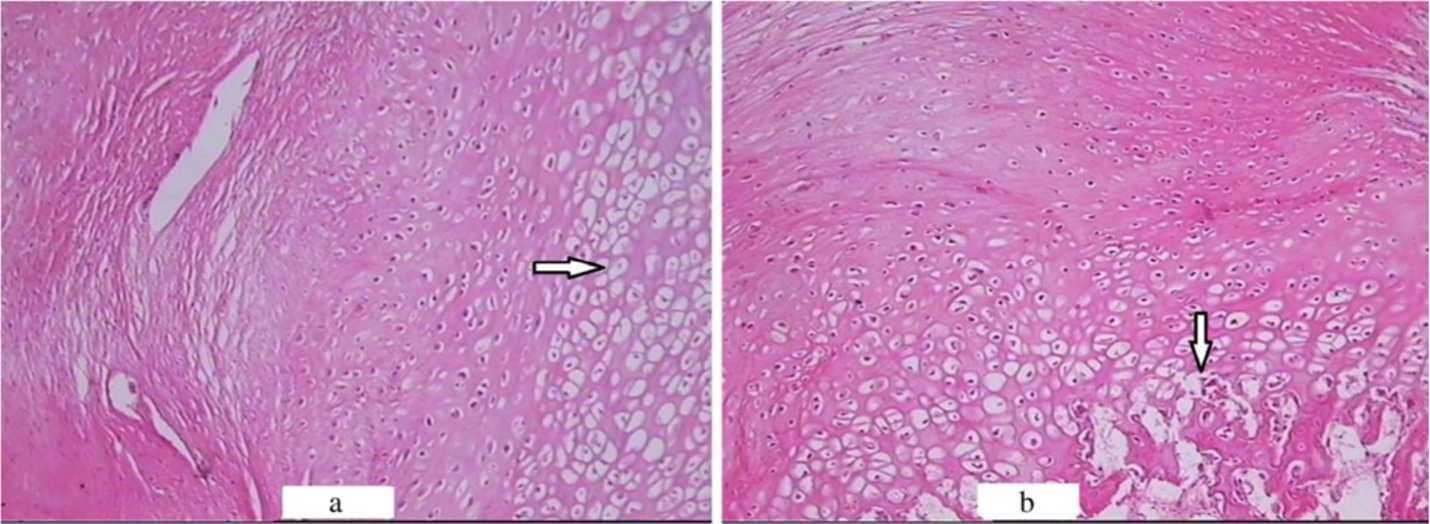
**HE staining of the callus in the saline-treated (a) and Sal B-treated (b) groups at week 3 post-fracture. (a)** Some cartilage cells(arrow) were seen in the saline-treated group at week 3 post-fracture (HE × 100). **(b)** Some bone matrix(arrow) accompanied with cartilage cells was seen in the Sal B-treated group at week 3 post-fracture (HE × 100).

**Figure 5:**
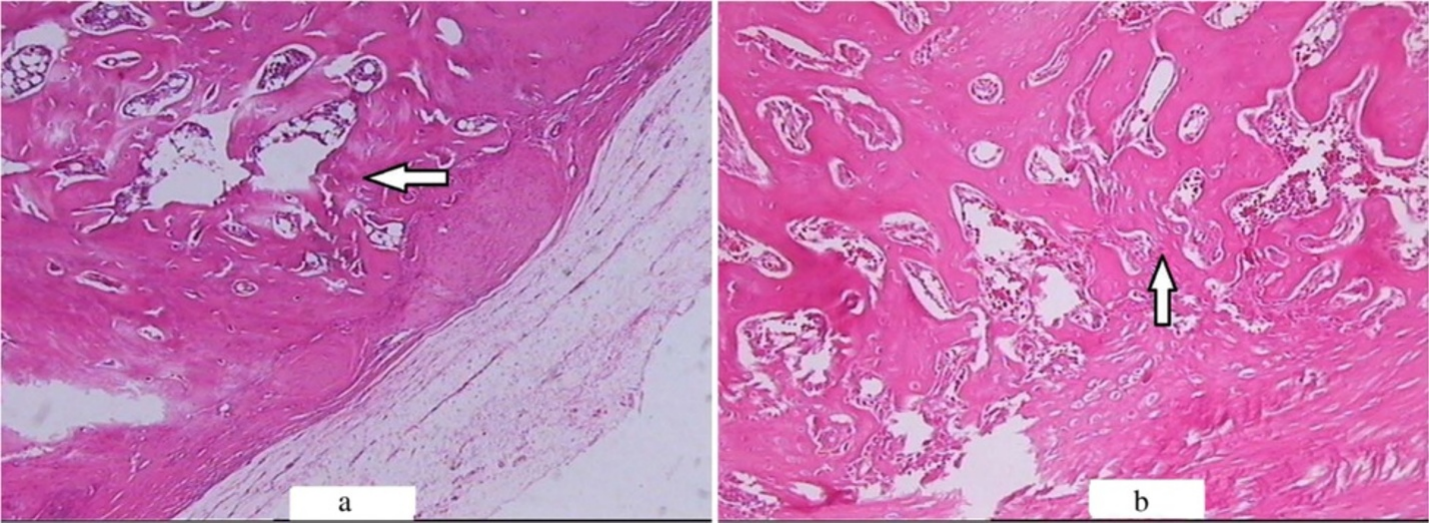
**HE staining of the callus in the saline-treated (a) and Sal B-treated (b) groups at week 8 post-fracture. (a)** Spongiosa(arrow) was seen at week 8 post-fracture in the saline-treated group (HE × 100). **(b)** More trabeculae(arrow) were observed and the spongiosa was more compact in the Sal B-treated group at week 8 post-fracture (HE × 100).

**Figure 6:**
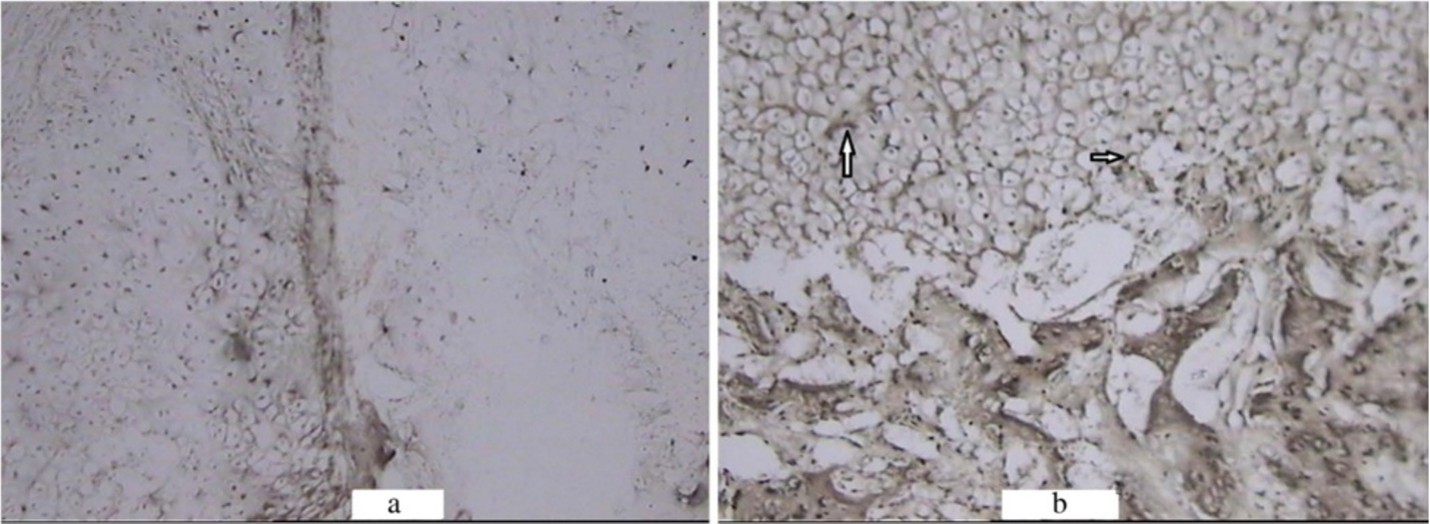
**ALP activity coloration of the callus in the saline-treated (a) and Sal B-treated (b) groups at week 1 post-fracture (×100).** Black precipitate (arrow) was more obvious in the experimental group.

**Figure 7:**
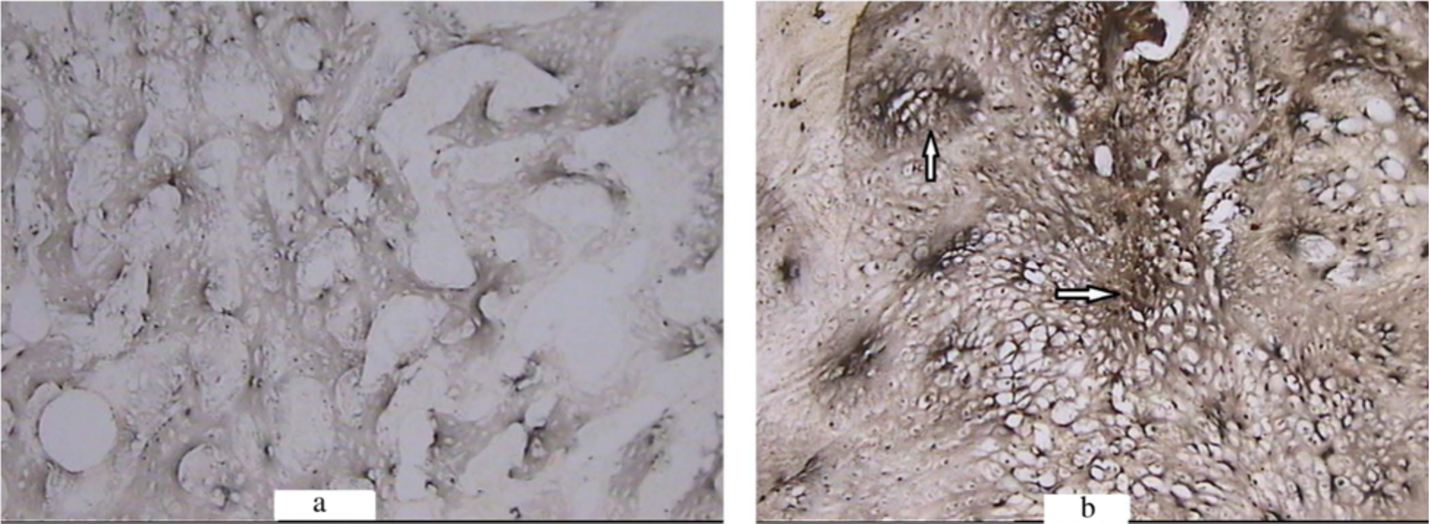
**ALP activity coloration of the callus in the saline-treated (a) and Sal B-treated (b) groups at week 3 post-fracture (×100).** ALP activity coloration was more obvious in the experimental group. Black precipitate was especially prominent around the cartilage cells (arrow).

**Figure 8:**
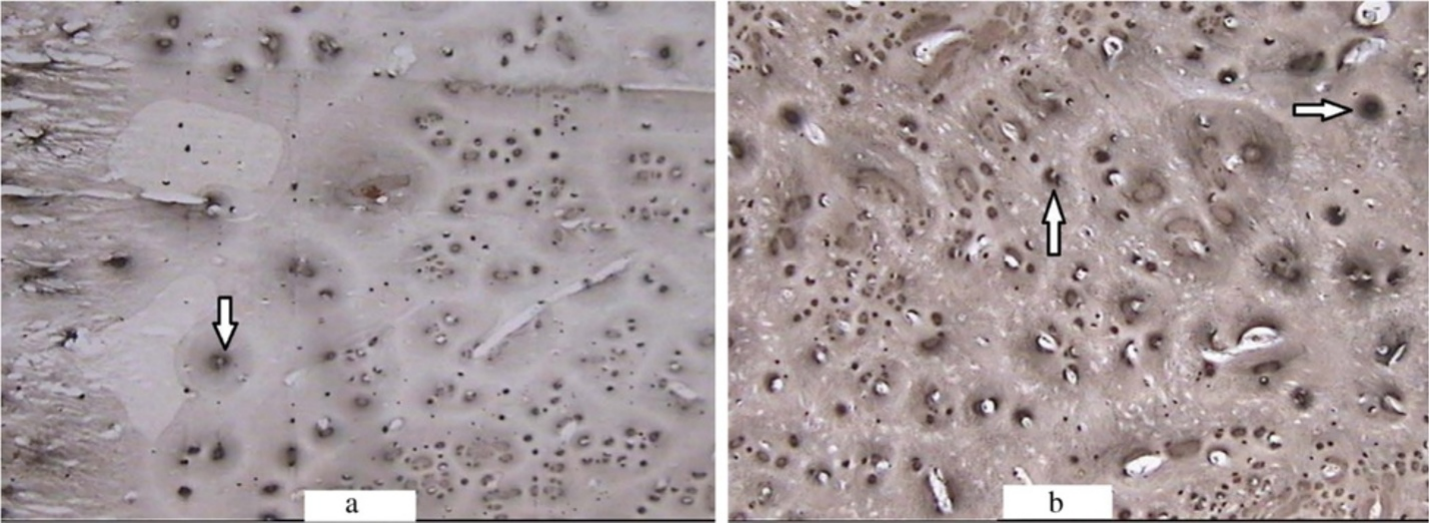
**ALP activity coloration of the callus in the saline-treated (a) and Sal B-treated (b) groups at week 8 post-fracture (×100).** Black precipitate (arrow) around bone lacunae was seen.

Histopathological scores were found to be significantly different between the control and experimental groups at weeks 1, 3 and 8 post-fracture (*P* < 0.05) (Table 4; Figures 3, 4 and 5). ALP coloration was more obvious in the experimental group at weeks 1 and 3 post-fracture (*P* < 0.05) (Table 5; Figures 6, 7 and 8).

**Table 4:** **Histopathological scores**: **median(min-max)**

Group	Week 1	Week 3	Week 8	Week 12
Saline Control	0 (0–1)	2.5 (2–3)	3 (3–4)	4 (3–4)
Sal B	1 (0–2)	3 (2–3)	4 (3–4)	4 (4–4)
P value	0.045	0.022	0.019	0.317

Note: Value are median (min-max).

Table 4 shows that the histopathological scores were significantly higher in the Sal B-treated group than in the Saline-treated group at weeks 1, 3 and 8 post-fracture (*P* < 0.05).

**Table 5:** **Callus section image analysis after calcium cobalt staining**

Group	Week 1	Week 3	Week 8	Week 12
Saline Control	44.28 (25.05)	62.42 (37.86)	121.12 (63.69)	8.33 (4.08)
Sal B	107.31 (92.72)	131.34 (46.47)	123.75 (69.69)	9.67 (4.16)
P value	0.033	0.001	0.957	0.660

Note: Value are means (SD).

Table 5 shows the results of the slice image analysis after staining. ALP level in the Sal B-treated group was significantly higher than in the saline-treated group at weeks 1 and 3 post-fracture (*P* < 0.05).

In the present study, we investigated the effects of Sal B on fracture healing in rats. First, our results showed that Sal B could accelerate callas mass growth. Second, we found that Sal B could promote the process of bone formation. Third, our experimental results revealed that Sal B might stimulate ALP activity during early fracture healing.

**Figure 9:**
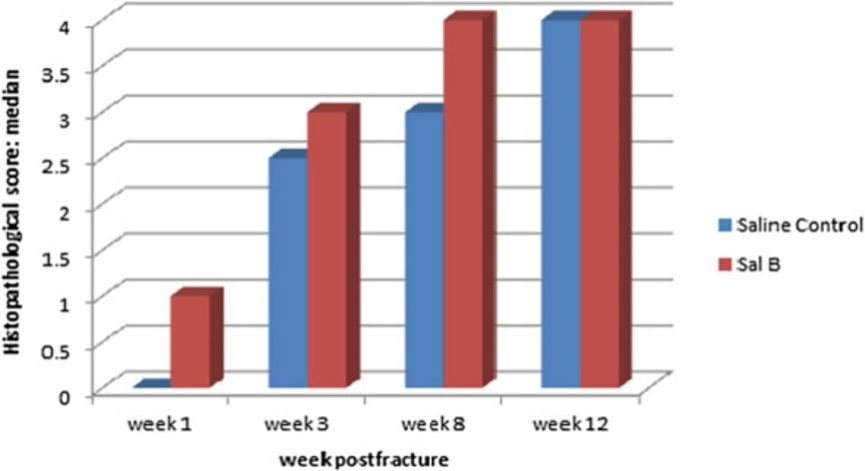
**Bar graph showing that the histopathological scores in the Sal B-treated group were higher than in the saline-treated group, which were significantly different at weeks 1, 3 and 8 post-fracture.**

**Figure 10:**
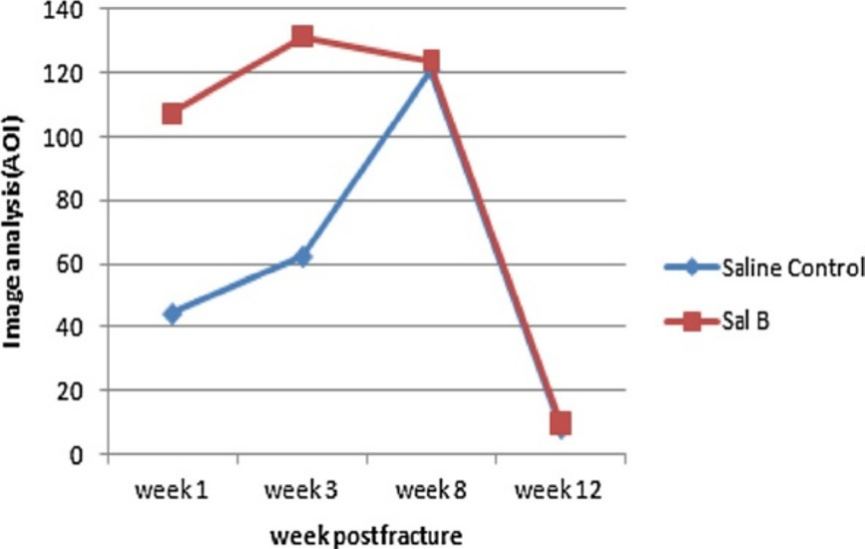
**Graph showing the ALP slice image analysis scores.** ALP levels in the Sal B-treated group were much higher than in the saline-treated group at weeks 1 and 3 post-fracture.

ALP plasma concentration of ALP is one biochemical indicator of bone formation; however, ALP can be derived from several tissues [23, 24]. Bone-specific ALP (BSAP) in the callus, which can be secreted by cartilage cells (Figure 3), is a more accurate index of osteoblastic activity than serum ALP. We found ALP level in the experimental group callus was significantly higher than in the control group at weeks 1 and 3 post-fracture (Figures 6 and 7). Correspondingly, the histological scores between the two groups were significantly different at weeks 1, 3 and 8 post-fracture (Figure 9). The HE stained sections showed that more cartilage cells were observed in the Sal B-treated group at week 1 post-fracture (Figure 3). And some bone matrix was seen at week 3 post-fracture in the Sal B-treated group (Figure 4). At week 8 post-fracture, we could observe more trabeculae and compacted spongiosa in the Sal B-treated group (Figure 5). These results suggest that Sal B could play a leading role in the promotion of BSAP activity and subsequently in bone formation during the early fracture healing process (especially at weeks 1 and 3 post-fracture) (Figure 10). Previous research *in vitro* has shown that Sal B stimulates increased ALP activity and osteocalcin secretion in a time- and dose-dependent manner [20]. However, we further demonstrated this same effect of BSAP in the local callus of our animal fracture model. Although there was no significant difference in ALP level between the control and experimental groups at week 8 post-fracture in this study (Figure 8), a numerically higher ALP level was observed in the experimental group compared with the control group. Fracture healing is a gradual and sequential process, whereby ALP is secreted leading to bone remodeling. We believe this may be the reason why there was no significant difference in ALP level at week 8 post-fracture, whilst the histological score in the experimental group was significantly greater.

Various factors are being studied for the purpose of accelerating fracture healing. Similar to the formation of other tissues, ostegenesis is closely linked with vascularity [25]. According to previous studies, Sal B can improve blood hemorheology [26] and blood circulation [27]. We believe the impact of Sal B during fracture healing may relate not only to the increase of ALP activity, but also to its effect in ameliorateing vascular ischemia. One limitation of our study, therefore, was the lack of study into tissue vasculature. Beyond this, many other factors that are closely associated with the union process (such as BMP [28], IGF-1 [29], OPG and RANKL, to name a few) should be evaluated in future studies. Another limitation of our study was that we were unable to investigate every phase of bone healing (including weeks 2 and 4) owing to a lack of availability in the laboratory where we conducted our study. More specific experiments should be performed for a better understanding of the effect of Sal B on bone healing.

## Conclusions

As a major water soluble component extracted from *Radix Salviae miltiorrhizae*, Sal B was demonstrated to be an effective component in fracture healing. Sal B accelerated levels of ALP in the callus, which in turn likely promoted the healing process. Our findings support further investigation of Sal B in stimulating osteogenesis as a potential therapeutic strategy, not only in trauma fractures but also other bone diseases.

## Authors’ information

Attending physician, Department of Traumatology, Shuguang Hospital Affiliated to Shanghai University of Traditional Chinese Medicine, Shanghai , China

Major degree: Master of Medicine

## Abbreviations

ALP: Alkaline phosphatase
Sal B: Salvianolic acid B
TCM: Traditional Chinese medicine
HE: Hematoxylin and eosin staining
BSAP: Bone-specific alkaline phosphatase
BMP: Bone morphogenetic protein
IGF-1: Insulin-like growth factor-1.

## References

[1] Zhou L, Zuo Z, Chow MS (2005). Danshen: an overview of its chemistry, pharmacology, pharmacokinetics, and clinical use. J Clin Pharmacol.

[2] Wu B, Liu M, Zhang S (2007). Dan Shen agents for acute ischaemic stroke. Cochrane Database Syst Rev.

[3] Wang C, Zhao X, Mao S, Wang Y, Cui X, Pu Y (2006). Management of SAH with traditional Chinese medicine in China. Neurol Res.

[4] Xing HC, Li LJ, Xu KJ, Shen T, Chen YB, Chen Y, Fu SZ, Sheng JF, Chen CL, Wang JG (2005). Effects of Salvia miltiorrhiza on intestinal microflora in rats with ischemia/reperfusion liver injury. Hepatobiliary Pancreatol Dis Int.

[5] Chen CG, Wang YP (2006). Magnesium lithospermate B ameliorates renal cortical microperfusion in rats. Acta Pharmacol Sin.

[6] Hoffmann SC, Kampen RL, Amur S, Sharaf MA, Kleiner DE, Hunter K, John Swanson S, Hale DA, Mannon RB, Blair PJ (2002). Molecular and immunohistochemical characterization of the onset and resolution of human renal allograft ischemia-reperfusion injury. Transplantation.

[7] Bando Y, Tsukamoto Y, Katayama T, Ozawa K, Kitao Y, Hori O, Stern DM, Yamauchi A, Ogawa S (2004). ORP150/HSP12A protects renal tubular epithelium from ischemia-induced cell death. FASEB J.

[8] Chen Y, Ruan Y, Li L, Chu Y, Xu X, Wang Q, Zhou X (2003). Effects of Salvia miltiorrhiza extracts on rat hypoxic pulmonary hypertension, heme oxygenase-1 and nitric oxide synthase. Chin Med J (Engl).

[9] Reignier J, Sellak H, Lemoine R, Lubineau A, Mazmanian GM, Detruit H, Chapelier A, Herve P (1997). Prevention of ischemia-reperfusion lung injury by sulfated Lewis(a) pentasaccharide. The Paris-Sud University Lung Transplantation Group. J Appl Physiol.

[10] Shi W, Fu S, Du N (2000). Effect of effective fraction of Radix Salviae Miltiorrhizae on procollagen gene expression in fracture healing. Zhongguo Zhong Xi Yi Jie He Za Zhi.

[11] Zhang JY (1984). Effect of Salvia miltiorrhiza root on calcium deposition in experimental fracture healing. Zhong Xi Yi Jie He Za Zhi.

[12] Chan K, Chui SH, Wong DY, Ha WY, Chan CL, Wong RN (2004). Protective effects of Danshensu from the aqueous extract of Salvia miltiorrhiza (Danshen) against homocysteine-induced endothelial dysfunction. Life Sci.

[13] Kamata K, Iizuka T, Nagai M, Kasuya Y (1993). Endothelium-dependent vasodilator effects of the extract from Salviae Miltiorrhizae radix. A study on the identification of lithospermic acid B in the extracts. Gen Pharmacol.

[14] Hu P, Luo GA, Zhao Z, Jiang ZH (2005). Quality assessment of radix salviae miltiorrhizae. Chem Pharm Bull (Tokyo).

[15] Lam FF, Yeung JH, Chan KM, Or PM (2007). Relaxant effects of danshen aqueous extract and its constituent danshensu on rat coronary artery are mediated by inhibition of calcium channels. Vasc Pharmacol.

[16] Li YG, Song L, Liu M, Hu ZB, Wang ZT (2009). Advancement in analysis of Salviae miltiorrhizae Radix et Rhizoma (Danshen). J Chromatogr A.

[17] Ho JH, Hong CY (2011). Salvianolic acids: small compounds with multiple mechanisms for cardiovascular protection. J Biomed Sci.

[18] Ji XY, Tan BK, Zhu YZ (2000). Salvia miltiorrhiza and ischemic diseases. Acta Pharmacol Sin.

[19] Zhong J, Tang MK, Zhang Y, Xu QP, Zhang JT (2007). Effect of salvianolic acid B on neural cells damage and neurogenesis after brain ischemia-reperfusion in rats. Yao Xue Xue Bao.

[20] Cui L, Li T, Liu Y, Zhou L, Li P, Xu B, Huang L, Chen Y, Tian X, Jee WS (2012). Salvianolic acid B prevents bone loss in prednisone-treated rats through stimulation of osteogenesis and bone marrow angiogenesis. PLoS One.

[21] Bonnarens F, Einhorn TA (1984). Production of a standard closed fracture in laboratory animal bone. J Orthop Res.

[22] Allen HL, Wase A, Bear WT (1980). Indomethacin and aspirin: effect of nonsteroidal anti-inflammatory agents on the rate of fracture repair in the rat. Acta Orthop Scand.

[23] Oni OO, Stafford H, Gregg PJ (1989). An experimental study of the patterns of periosteal and endosteal damage in tibial shaft fractures using a rabbit trauma model. J Orthop Trauma.

[24] Moss DW (1987). Diagnostic aspects of alkaline phosphatase and its isoenzymes. Clin Biochem.

[25] Viateau V, Guillemin G, Yang YC, Bensaid W, Reviron T, Oudina K, Meunier A, Sedel L, Petite H (2004). A technique for creating critical-size defects in the metatarsus of sheep for use in investigation of healing of long-bone defects. Am J Vet Res.

[26] Yang Q, Wang S, Xie Y, Wang J, Li H, Zhou X, Liu W (2010). Effect of salvianolic Acid B and paeonol on blood lipid metabolism and hemorrheology in myocardial ischemia rabbits induced by pituitruin. Int J Mol Sci.

[27] Pan C, Lou L, Huo Y, Singh G, Chen M, Zhang D, Wu A, Zhao M, Wang S, Li J (2011). Salvianolic acid B and tanshinone IIA attenuate myocardial ischemia injury in mice by NO production through multiple pathways. Ther Adv Cardiovasc Dis.

[28] Westerhuis RJ, van Bezooijen RL, Kloen P (2005). Use of bone morphogenetic proteins in traumatology. Injury.

[29] Schmidmaier G, Wildemann B, Heeger J, Gabelein T, Flyvbjerg A, Bail HJ, Raschke M (2002). Improvement of fracture healing by systemic administration of growth hormone and local application of insulin-like growth factor-1 and transforming growth factor-beta1. Bone.

[CR30] The pre-publication history for this paper can be accessed here:http://www.biomedcentral.com/1472-6882/14/493/prepub

